# COPING STRATEGIES AMONG FAMILY CAREGIVERS FOR OLDER ADULTS WITH SERIOUS MEDICAL ILLNESS: A SCOPING REVIEW

**DOI:** 10.1093/geroni/igae098.2383

**Published:** 2024-12-31

**Authors:** Ellen Csikai, Quentin Maynard, Marina Olmos, M D Sarafat Hossain

**Affiliations:** University of Alabama, Tuscaloosa, Alabama, United States; University of Southern Indiana, Evansville, Indiana, United States; University of Murcia, Murcia, Murcia, Spain; University of Alabama, Tuscaloosa, Alabama, United States

## Abstract

Family caregivers often provide many hours of care for years frequently leading to reduced abilities to cope with the care and quality of life for the caregivers (Lewis, et al, 2019). Family caregivers who provide care to older adults near the end of life face even greater challenges, including increased physical and emotional burden. The aim of this study was to identify coping strategies used by family caregivers of older adults with serious medical illness that help them manage in the caregiving role. A scoping literature review of qualitative and quantitative research (published in peer-review journals since 2012) involving community-dwelling family caregivers of older adults with serious medical illness, near end of life, was conducted. A total of 426 articles met the search criteria. After initial systematic screening of titles/abstracts, followed by full text review, through consensus meetings, eight (n=8) articles met inclusionary criteria and were included in the review. Among the studies, six were qualitative; two quantitative. Samples varied in medical diagnoses; sizes ranging 8-228. Shared results regarding coping strategies were how various coping strategies were used to manage both physical and emotional challenges. Problem-solving and emotion-focused coping types were common along with social support by relying on others. Family caregivers of older adults with serious illness must cope daily with challenges. With better understanding of current coping strategies used to manage physical and emotional demands, professionals can recognize and intervene to bolster coping abilities and assist with resources to safeguard caregivers’ well-being.

